# Epidemiological Features of Injured Patients Examined by Tehran Emergency Medical Service Technicians

**DOI:** 10.22114/ajem.v0i0.198

**Published:** 2019-06-25

**Authors:** Peyman Saberian, Amir Reza Farhoud, Parisa Hasani-Sharamin, Maryam Moghaddami, Fatemeh Keshvari

**Affiliations:** 1. Pre-Hospital and Hospital Emergency Research Center, Tehran University of Medical Sciences, Tehran, Iran.; 2. Department of Anesthesiology, Imam Khomeini Hospital Complex, Tehran University of Medical Sciences, Tehran, Iran.; 3. Joint Reconstruction Research Center, Department of Orthopedic Surgery, Imam Khomeini Hospital Complex, Tehran University of Medical Sciences, Tehran, Iran.; 4. Tehran Emergency Medical Service Center, Tehran, Iran.; 5. National Emergency Medical Service Organization, Tehran, Iran.

**Keywords:** Emergency Medical Services, Epidemiologic Studies, Wounds and Injuries

## Abstract

**Introduction:**

Knowledge of epidemiological aspects can be a useful guide in determining the resources for better prevention and management of injuries. There are some performed studies on this topic in Iran, based on the limited hospital database. However, to the best of our knowledge, there is not any survey based on the pre-hospital database

**Objective:**

The purpose of this study was to assess baseline characteristics of the traumatic patients according to the records of Tehran Emergency Medical Service (EMS) Center to present descriptive statistics of their epidemiological features.

**Method:**

This cross-sectional study was conducted retrospectively, using Tehran EMS center data registry. All traumatic patients examined by EMS in Tehran, Iran following call to emergency medical dispatcher were included. By reviewing the EMS technicians’ mission forms, required data were extracted. The mission form contains information such as age, sex, injured location, damage mechanism, accident location (home, workplace, street), time of call, the outcome of the patient’s ambulance mission and the results of the assessment of the technician, etc.

**Results:**

Totally, 56612 injured cases with the mean age of 33.1±15.6 years were examined by EMS during one-year study period of whom 80.4% were male. Crude Incidence Rate was 10.5 and 2.5 per 1000 in male and female, respectively. Traffic accident and then fall were the two most prevalent mechanism of injuries. All types of injuries were significantly more prevalent in males (P<0.001). Most injuries were in winter season with 15570 cases (27.5%). Car accident was prevalent in winter and other injuries were significantly prevalent in spring (P<0.001). The most frequent places of injuries occurred on main roads and streets (55.7%). All of the road-related injuries was prevalent in winter, whereas injuries in other places were prevalent in spring (P<0.001). Most of the cases (78.3%) were transferred to the health centers, but 20.7% did not consent to treatment and transmission. Only 222 cases (0.4%) died, that 95% was due to traffic accident. there was a significant relationship between the number of injured organs and the death; So that the highest death rate occurred for those with more than 5 injured organs (P <0.001).

**Conclusion:**

Based on the findings, traffic accident was the most frequent cause of trauma that led to visiting a traumatic patient by an EMS technician in Tehran, Iran. Injuries in all age groups were more prevalent in males, and the involvement of 5 or more injured organ had a significant relationship with mortality.

## Introduction

Today, trauma is one of the major causes of death, with almost 5.8 million people worldwide losing their lives each year; and it is anticipated that by the year 2020, it could reach 8.4 million per year (1–5). In addition, injuries are considered as a major source for loss of medical expenses and health budgets. For example, the direct cost of injuries accounts 5% of the health care budget in the Netherlands; in Spain the overall cost of road traffic crashes (RTC) represents 1.35% of the gross national product; and in Iran total direct and indirect costs of a traumatic patient following RTC were reported equal to 1000$ in 2016 (6–8). The incidence of trauma is higher in low-income and middle-income countries, which do not have the capacity to deal with and suffer from lack of necessary facilities for trauma management; In result, injuries frequently lead to disability or death that imposes significant costs on the injured family and the health system.

Knowledge of epidemiological aspects can be a useful guide in determining the resources for better prevention and management of injuries. There are some performed studies on this topic in Iran, based on the limited hospital database (9–11). However, to the best of our knowledge, there is not any survey based on the pre-hospital database. The purpose of this study was to assess baseline characteristics of the traumatic patients based on records of Tehran Emergency Medical Service (EMS) Center to present descriptive statistics of their epidemiological features.

## Methods

### Study design and setting

This cross-sectional study was conducted retrospectively, using Tehran EMS center data registry. Ethical approval was received from the ethical committee of Tehran University of Medical Sciences (IR.TUMS.VCR.REC.1397.969).

All information was used anonymously. The principles of ethics and preservation of confidentiality of information were respected by the authors.

### Study Population

All traumatic patients examined by EMS in Tehran, Iran following call to emergency medical dispatcher were included. No exclusion criteria were considered. Sampling was done by census method from March 21, 2017, to March 20, 2018.

### Data gathering

For this propose a predetermined checklist was used and the investigators by reviewing EMS technicians’ mission forms, extracted required data. The mission form contains information such as age, sex, injured location, damage mechanism, accident location (home, workplace, street), contact time of call, the outcome of the patient’s ambulance mission and the results of the assessment of the technician, etc.

### Statistical analysis

For descriptive assessment, values were expressed as frequency (number and percentage), mean [standard deviation (SD)], median [interquartile range (IQR)]. Fisher’s exact test and chi-square tests were used for comparisons of categorical variables and independent t-test were was used to compare numerical variables. Kolmogorov-Smirnov test was used to check the normality assumption for the variable. All analysis of the studied relationships was performed with a 95% confidence Interval, so P-value<0.05 was considered statistically significant. Statistical analyses were performed using the SPSS software.

## Results

During one-year study period, 56612 injuries were recorded, out of which 45493 cases (80.4%) were in men and 11076 cases (19.6%) were in women (for 43 individuals the sex was undetermined). Crude Incidence Rate (CIR) was 10.5 and 2.5 per 1000 in male and female, respectively. The mean age of occurred injuries was 33.1 years (SD = 15.6 years) and the median was 30 (IQR=19) years. The mean age of injured in males was lower than in females (32.3±14.7 vs. 36.6±18.2), and this difference was statistically significant (p<0.001). The number of injuries was increased with age and peaked at ages 21 to 30 years (33.5%) and then declined. Injuries in all age groups were more prevalent in males, and the highest and the lowest ratio were seen in the group age of 11–20 and more than 70 years old, respectively (6.1 vs. 1.9) (P<0.001) (Table 1). Off all total, 95.2% were car accident and then fall was the most prevalent injury. All types of injuries were significantly prevalent in males (P<0.001) so that violence was 8.4 times and car accident 4.2 times higher in men (Table 1).

**Table 1:** Frequency and sex ratio of injury by Age group and type of injuries in studied patients

	**Male (% of total)**	**Female (% of total)**	**Sex ratio**	**Total (%)**
**Age group**				
				
0 – 10	758 (32.5)	1576 (67.5)	2.1	2334 (4.1)
11 – 20	1191 (14.2)	7220 (85.8)	6.1	8411 (14.9)
21 – 30	2727 (14.4)	16173 (85.6)	5.9	18900 (33.5)
31 – 40	2318 (18.9)	9956 (81.1)	4.3	12274 (21.8)
41 – 50	1592 (24.3)	4960 (75.7)	3.1	6552 (11.6)
51 – 60	1192 (27.8)	3096 (72.2)	2.6	4288 (7.6)
61 – 70	751 (35.1)	1390 (64.9)	1.9	2141 (3.8)
More than 70	501 (34.0)	972 (66.0)	1.9	1473 (2.6)

**Total**	**45343 (80.4)**	**11030 (19.6)**	**4.1**	**56373 (100)**

**Type of injuries**				
				
Car accident	43548 (80.9)	10304 (19.1)	4.2	53852 (95.2)
Fall	669 (61.8)	414 (38.2)	1.6	1083 (1.9)
Trauma and mechanical crash	622 (71.9)	243 (28.1)	2.3	865 (1.5)
Violence	489 (89.4)	58 (38.2)	8.4	547 (1.0)
Suicide and self-harm	27 (69.2)	12 (31.8)	2.3	39 (0.1)
Other	109 (78.4)	30 (21.6)	3.6	139 (0.2)
Unspecified	29 (65.9)	15 (34.1)	1.9	44 (0.1)

**Total**	**45493 (80.4)**	**11076 (19.6)**	**4.1**	**56569 (100)**

**Figure 1:**
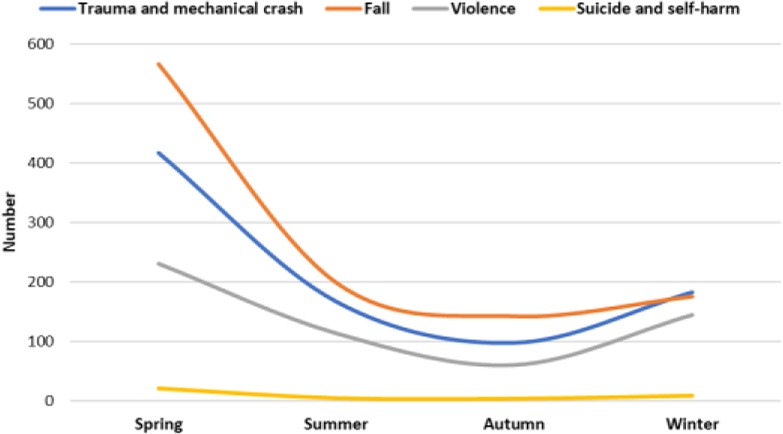
Comparison of trauma etiology in studied patients by season

Most injuries were in winter with 15570 cases (27.5%). car accident was prevalent in winter and other injuries were significantly prevalent in spring (P<0.001) (Table 2 and Figure 1). The most frequent places of injuries were on main road and street (55.7%), highway (18.4%) and alley (16.5%). All of the road-related injuries were prevalent in winter, whereas injuries in other places were prevalent in spring (P<0.001) (Table 1).

**Table 2:** Frequency of injuries based on type and place in studied patients

	**Spring (% of total)**	**Summer (% of total)**	**Autumn (% of total)**	**Winter (% of total)**	**Total**
**Type of injuries**					
					
Car accident	11937 (22.1)	13392 (24.8)	13542 (25.1)	15023 (27.9)	53852 (95.2)
Fall	566 (52.2)	199 (18.4)	143 (13.2)	176 (16.2)	1083 (1.9)
Trauma and mechanical crash	417 (48.2)	167 (18.4)	98 (11.3)	183 (21.2)	865 (1.5)
Violence	230 (42.0)	113 (20.7)	60 (11.0)	144 (26.3)	547 (1.0)
Suicide and self-harm	21 (53.8)	5 (12.8)	4 (10.3)	9 (23.1)	39 (0.1)
Other	71 (51.1)	25 (18.0)	19 (13.7)	24 (17.3)	139 (0.2)
Unspecified	13 (29.5)	11 (25.0)	9 (20.5)	11 (25.0)	44 (0.1)

**Place of injuires**					
					
Main road and street	6911 (21.9)	7607 (24.1)	8080 (25.6)	8921 (28.3)	31519 (55.7)
Highway	2266 (21.7)	2784 (26.7)	2595 (24.8)	2799 (26.8)	10444 (18.4)
Alley	2181 (23.4)	2317 (24.9)	2270 (24.4)	2546 (27.3)	9314 (16.5)
Residential	839 (33.7)	473 (19.0)	492 (19.8)	685 (27.5)	2489 (4.4)
Public and recreational place	345 (36.7)	248 (26.4)	148 (15.7)	199 (21.2)	940 (1.7)
Administrative and industrial	256 (31.4)	182 (22.3)	166 (20.4)	211 (25.9)	815 (1.4)
Other and Unspecified	457 (41.9)	301 (27.6)	124 (11.4)	209 (19.2)	1091 (1.9)

**Total**	**13255 (23.4)**	**13912 (24.6)**	**13875 (24.5)**	**15570 (27.5)**	**56612 (100)**

Among studied patients, 28916 cases (51.1%) suffered blunt trauma, and 8458 (14.9%) penetrating trauma. In 19238 cases (34.0%), blunt/penetrating trauma classification (34.0%) was not specified by the EMS technicians in their mission form.

**Figure 2:**
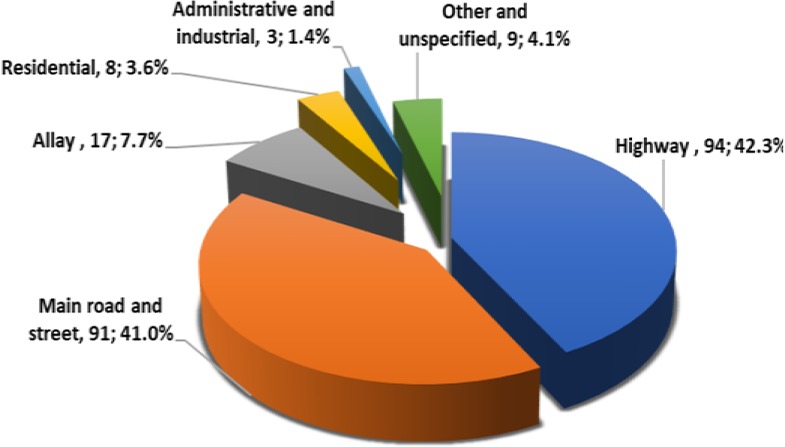
Location of trauma occurrence in dead cases

Most cases (78.3%) have been transferred to the health center and 20.7% did not consent to get treatment and transmission. Only 222 cases (0.4%) died, that 95% was due to car accident; 2.3% and 1.4% were due to trauma and fall, respectively. Most of the dead cases (91.0%) occurred in highway, main road and street and alley, and then in residential place (3.6%) (Figure 2) (P<0.001).

The frequency of injured organ in studied patients based on anatomy is reported in table 3. There were 17418, 8467, 19572 and 29369 recorded injuries on head and neck, torso, upper and lower limbs respectively. Among studied patients, 20526 cases (36.3%) were reported as multiple trauma. The number of injured organs based on the patients’ mortality was reported in table 4. In the majority of cases, only one (62.9%) or two limbs (22.5%) were involved, and there was a significant relationship between the number of injured organs and death; So that the highest death rate occurred for those with more than 5 injured organs (P <0.001).

**Table 3:** Frequency of organ injuries in studied patients based on anatomy

**Organ**	**Number**	**%**
**Head**	14715	26.0
**Neck**	2703	4.8
**Torso**		
Abdomen and flank	2504	4.4
Chest	1808	3.2
Pelvic	1578	2.8
Waist	1301	2.3
Spine	1043	1.8
Genitalia	233	0.4
**Upper limb**		
Shoulder	4781	8.4
Hand	4696	8.3
Forearm	3337	5.9
Elbow	2828	5.0
Wrist	2818	5.0
Arm	1112	2.0
**Lower limb**		
Knee	8783	15.5
Leg	6332	11.2
Ankle	6105	10.8
foot	5596	9.9
Thigh	2553	4.5
**Unknown**	1329	2.3

**Table 4:** The number of injured organs based on the patients’ mortality

**Number**	**Live (n=56390) (% of total)**	**Dead (n=222) (% of total)**	**Total (n=56612) (100%)**
**One**	34580 (99.5)	177 (0.5)	34757 (61.4)
**Two**	12452 (99.9)	12 (0.1)	12464 (22.0)
**Three**	5153 (99.8)	9 (0.2)	5162 (9.1)
**Four**	1857 (99.8)	3 (0.2)	1860 (3.3)
**Five**	690 (99.9)	1 (0.1)	691 (1.2)
**More than five**	345 (98.9)	4 (1.1)	349 (0.6)
**Undetermined**	1313 (98.8)	16 (1.2)	1329 (2.3)

## Discussion

### • Sampling

Census sampling method based on the prehospital database had been applied in the current study and during one-year study period, 56612 consecutive recorded cases were enrolled which is the strong point of this study in comparison with previous ones. Most of the previous studies had been performed based on the hospital database, so they had less sample size like the one conducted by Hashemi et al during 2012 in Iran using cluster sampling method that gathered the information of 8626 patients (11); or the one performed by Byun et al in South Korea by census sampling which assessed 17007 cases (12).

### • Sex

Like other studies performed on nonspecific population, most of the cases were male (80.4%) and CIR in male and female was 10.5 and 2.5 per 1000, respectively. The mean age of male patients was significantly lower than female ones; As well, all type of injuries were also significantly more prevalent in males than females, so that violence was 8.4 times and car accident was 4.2 times higher in males than in females. Injuries in all age groups were more prevalent in males than females in the current study; unlike Hashemi et al who reported that after 40-year-old, women have a higher risk of injuries compared with men (11).

### • Age

The mean age of injured patients was 33.1±15.6 years and as mentioned before the mean age of males was significantly lower than females. The number of injured was increased with age and peaked at 21–30 years and then declined. As previously mentioned, the number of injuries in all age groups was more prevalent in males than in females. The highest and the lowest ratio were seen in the group age of 11–20 years and more than 70 years old, respectively. Age is an effective factor on the type of traumatic events; although young and middle-aged people are more likely to be traumatized than others, Some types of trauma like fall are more likely to happen in the elderly population. Age also has a relationship with injury severity resulting from a traumatic event, so that two patients at different ages may experience different injuries with varying severity from a similar event (13–16).

### • level of consciousness

This is an important prognostic factor that must be evaluated by an EMS technician on all traumatic patients on the scene and must be repeated at least once again on arrival to the emergency department. There are various methods including Glasgow coma score (GCS) or Full Outline of Unresponsiveness (FOUR) score in this regards; It is likely that these scoring systems had the same predictive value for the outcome of multiple trauma patients (17, 18).

### • Anatomy

Overall, lower limbs were affected more than other parts of the body whereas torso was the least affected site in the studied patients in the current study, and almost one-third of the cases were reported as multiple trauma. In line with our results, Jha et al in India, Ramouz et al in Iran and also Byun et al in South Korea also reported limbs as the most affected part of the body in hospitalized traumatic patients who had mainly injured from blunt trauma (12, 19, 20). In another study performed by Thanni et al at a Nigerian teaching hospital Torso was reported as the least affected place following traumatic events that the results were along with our results (21). On the contrary to the results of these studies, Mahdian et al that conducted an epidemiologic study on hospitalized traffic accident victims, reported that head was the most frequently affected place (22).

### • Mechanism

Hashemi et al reported that most cases (96.6%) were caused following unintentional injuries (11), and only 1.1% of studied patients were affected by violence or self-injury. Although the type of trauma was not recorded by the EMS technicians in one-third of the cases, half of them suffered from blunt trauma, Byun et al had reported similar results (12). Most of the available resources extracted from epidemiological studies have identified traffic accidents as the most common mechanism of traumatic incidences (20, 21); like what we have concluded in the study.

### • Place

The most common place where the incidents occurred were main roads and streets, highways and alleys respectively. Perhaps this statistic can be justified by the fact that the largest volume of daily traffic is in the main streets of the city, so the greater the volume of traffic in circulation, the greater the chance of accidents.

### • Season

Considering the significant role of traffic accidents in trauma, a little more than a quarter of the studied cases, in overall, was in winter; But car accident was more prevalent in winter and other injuries were significantly more prevalent in spring. It also should be mentioned that all of the main road and street related injuries were more prevalent in winter, whereas injuries in other places were more prevalent in spring. The snow and rain lead to slippery streets in late autumn and winter as well as shorter duration of daylight can be mentioned as effective factors in this regard. Mehrpour et al described epidemiological aspects of orthopedic injuries of 18890 adult patients in a tertiary trauma center in Iran. They reported that most of the orthopedic injuries had occurred during February and March and the least belonged to June (23). Their results are in line with the current study. As well, Peymani et al performed an epidemiological study to assess characteristics of fatal pedestrian accidents in Fars Province of Iran and found that most of the injuries had occurred during late summer (September) (24).

### • Outcome

Less than 1% of the studied cases died, that 95% was due to car accidents. Most of the death cases occurred in the highway, road and alley and then in the residential place. There was a significant relationship between the number of injured organs and death in the current study and the highest death rate occurred for those with more than 5 injured organs. The same correlation has not been reported in available literature yet. Byun et al reported their mortality rate as 5.6% that apparently out of hospital deaths following trauma were not taken into account. They reported age ≥ 55 years, injury severity score ≥ 16, major head injury, cardiopulmonary resuscitation on admission as predictors of trauma mortality (12). Morrison et al assessed 13100 cases with fatal injuries during one decade in Scotland and found out that 36.3% of death cases occurred in the pre-hospital setting that mostly were related to traffic accidents, but most of the in-hospital deaths resulted from fall on the same level. They also reported that the mean age of the pre-hospital dead cases was significantly lower than the hospital ones (25).

### Limitations

Lack of hospital follow up is the most important limitation of this study. Additive information from hospital management of studied patients would be valuable. Radiological findings, duration of hospitalization, therapeutic interventions, and the final outcome of the patients certainly increases the quality of such studies. Although the place of trauma was mentioned in the technicians’ mission forms, but if the severity of trauma in that point was recorded, would be helpful.

## Conclusions

Based on the findings, traffic accident was the most frequent cause of trauma that leads to being examined by EMS technicians in Tehran, Iran. There are more injured in all age groups of males, and the involvement of 5 or more injured organs had a significant relation with mortality.
